# A Cross-Sectional Analysis of Toothache Prevalence and Its Contributing Factors in Dental Outpatients

**DOI:** 10.7759/cureus.87115

**Published:** 2025-07-01

**Authors:** Lalitkumar P Gade, Harish K Khamkar, Abhijeet Kamble, Rashmi G Choudhary, Pranali S Patil, Vidya M Rodge

**Affiliations:** 1 Department of Oral Pathology, Smt. Mathurabai Bhausaheb Thorat Dental College and Hospital, Sangamner, IND

**Keywords:** dental, oral health, prevalence, risk factors, toothache

## Abstract

Introduction: This study aimed to determine the prevalence of toothaches in patients attending the outpatient department (OPD) of a dental institution in India. The primary objective was to identify the clinical and behavioral factors associated with toothaches to support the development of targeted preventive and treatment strategies for this population.

Materials and methods: A cross-sectional study was conducted over six months in the OPD of a dental college. Using consecutive sampling, 6732 patients were evaluated. Data were collected through a structured questionnaire and clinical examinations conducted by trained dental professionals. Information on demographic characteristics, oral hygiene habits, lifestyle behaviors (smoking, alcohol consumption, and tobacco use), and socioeconomic status was gathered. Clinical indicators including dental caries and periodontal status were also assessed. Bivariate analysis (chi-square test) and multivariate logistic regression were used to determine the associations between toothaches and potential risk factors. Point-biserial correlation was used to evaluate etiological factors. The odds ratio (OR) was calculated.

Results: Toothaches were reported in 3883 patients (58%). The prevalence of toothaches was significantly influenced by age, with patients aged 17-60 years showing higher odds (OR = 1.52, p = 0.023) than those under 17 years. Female patients were more than male patients (p < 0.001). Non-smokers (OR = 2.36, p = 0.021), non-tobacco users (OR = 1.98, p = 0.012), and non-alcohol users (OR = 2.12, p = 0.013) had higher odds of toothaches, indicating potential reverse causality. Poor oral hygiene practices, such as infrequent brushing (OR = 1.05, p = 0.001) and lack of dental floss use (OR = 1.96, p = 0.001), were associated with toothache. Urban residence (OR = 1.5, p = 0.001), higher education level, and low income emerged as significant predictors. Etiological analysis showed that self-medication had the strongest correlation with various causes of toothaches, including caries, trauma, and periodontitis.

Conclusion: The prevalence of toothaches was reported to be 58%. The findings highlight the multifactorial nature of toothache, with strong links to age, sex, oral hygiene habits, lifestyle behaviors, and socioeconomic status. Comprehensive public health strategies focusing on education, early intervention, and behavioral modification are essential for reducing the burden of toothaches in the population.

## Introduction

A toothache is one of the most common dental complaints, affecting millions of individuals worldwide and significantly affecting their quality of life [1]. It is often a manifestation of underlying oral diseases, including dental caries, pulpitis, periodontal disease, and abscesses, which are associated with varying degrees of pain and discomfort [2,3]. Although a toothache is a symptom, it serves as an important clinical marker of oral health status and unmet dental care needs [4]. In developing countries, such as India, where oral health often receives less attention in public health discourse, the prevalence of toothaches remains a substantial concern [5]. Addressing this issue requires a thorough understanding of the epidemiological burden and the associated risk factors.

Previous studies have indicated that toothaches are influenced by a wide range of clinical and behavioral factors [3,6]. Poor oral hygiene practices, such as infrequent brushing and neglect of dental checkups, significantly contribute to the development of dental caries and periodontal disease, which are the primary causes of toothaches [3,6]. Additionally, lifestyle factors such as tobacco use, smoking, and a high-sugar diet exacerbate the risk of dental problems, further contributing to the onset of toothaches [7,8]. Socioeconomic factors, including low income and limited access to dental care, also play a pivotal role in the persistence and severity of toothache, especially in rural and underserved populations [9].

The reported prevalence of toothaches ranges from 5 to 33% among countries like Norway, Brazil, and India [10]. Despite the high burden of oral diseases, epidemiological data on the prevalence of toothaches in India, particularly among patients seeking care in dental outpatient settings, are limited. Understanding the patterns of toothaches and their associated risk factors in this population is crucial for formulating targeted prevention and intervention strategies. Furthermore, dental colleges, as centers of both education and patient care, offer a unique setting in which to study these patterns, as they provide a diverse patient base, ranging from urban to rural populations.

This study aimed to evaluate the prevalence of toothaches among patients attending the outpatient department (OPD) of a dental college in India, and to identify the associated risk factors contributing to this condition. By examining both clinical and behavioral factors, this study sought to provide a comprehensive understanding of the underlying causes of toothaches in a dental OPD setting.

## Materials and methods

Study design

This cross-sectional study was conducted in the OPD of the Department of Oral Pathology, Smt. Mathurabai Bhausaheb Thorat Dental College and Hospital, Sangamner, India on patients attending the OPD over a period of six months, from January 2022 to June 2022. A consecutive sampling method was employed in which all patients who visited the OPD during the study period were included, ensuring comprehensive coverage of the population. This method provides a census of patients and eliminates the need for random sampling or sample-size estimation. The study population included all patients who presented for dental care, regardless of their specific complaints, and each patient was classified into two categories: those with and without a toothache. Ethical approval for the study was obtained from the Institutional Ethics Committee (EC/NEW/INST/2021/1810) and patient confidentiality was maintained throughout the research process. Written informed consent was obtained from all the patients, and no identifying information was used in the final analysis to ensure privacy. This study was conducted in accordance with the principles of the Declaration of Helsinki.

Inclusion and exclusion criteria

The inclusion criteria for the study required patients to be of any age or sex, attend the OPD for dental concerns, and be willing to provide written informed consent. Patients with dental or oral health-related complaints were eligible for inclusion, whereas those with nondental causes of facial pain, such as temporomandibular joint disorders or facial trauma, patients with systemic conditions that could affect oral health (such as uncontrolled diabetes), those on medications that influence dental outcomes (such as bisphosphonates), and those who were unable to provide complete information or refused to provide consent were excluded from the study.

Data collection

Data collection involved a standardized clinical examination and structured questionnaire administered by trained dental professionals. The questionnaire collected demographic information including age, sex, and socioeconomic status, as well as data on oral hygiene practices (frequency of brushing and use of dental floss) and lifestyle factors (smoking, tobacco use, and diet). Detailed medical and dental histories were obtained to assess previous treatments and overall oral health. The clinical examination focused on identifying the presence of toothache, defined as pain localized to one or more teeth, as well as assessing oral health indicators, such as dental caries, periodontal disease, and decayed or missing teeth.

Statistical analysis

The data were analyzed using IBM SPSS Statistics for Windows, Version 23 (Released 2015; IBM Corp., Armonk, New York, United States). Descriptive statistics were calculated to determine the prevalence of toothache and summarize the demographic characteristics. The normality of the data was assessed using the Shapiro-Wilk test, and the data were found to be normally distributed. To explore the association between toothache and potential risk factors, bivariate analyses were conducted using chi-square tests for categorical variables. A multivariate logistic regression model was built to identify independent risk factors associated with toothache, adjusting for confounding variables, such as age, sex, and oral hygiene practices. Odds ratios (ORs) with 95% confidence intervals (CIs) were calculated for each of the significant risk factors. Point-biserial analysis was used to assess the etiological factors of toothaches. A p-value less than 0.05 was considered statistically significant for all analyses.

## Results

In this study of 6732 patients, demographic factors related to the presence of toothaches were analyzed using the chi-square test. Age significantly influenced toothache occurrence, with 1221 (57%) patients under 17 years and 1946 (61%) of those between 17 and 60 years reporting toothache (p = 0.0001). Sex also showed a significant relationship (p = 0.0001), as a greater number of female patients experienced toothaches than male patients. Dietary habits were not significantly associated (p = 0.515), whereas smoking and tobacco use were significantly associated, with 1144 (63%) smokers (p = 0.0001) and 1539 (67%) tobacco users (p = 0.000). Similarly, alcohol consumption was impactful, with 1036 (73%) users reporting toothaches (p = 0.000). Brushing frequency and dental floss usage were significantly associated, as toothaches were more common among those brushing once daily (p = 0.000) and non-flossers (p = 0.045). Lower annual income, rural residence, illiteracy, self-medication, and rural residence were also significantly correlated with toothaches (p = 0.0001). The prevalence of toothaches was 58% (Table 1).

**Table 1:** Association of demographic factors with occurrence of toothaches using the chi-square test of association. *p-value < 0.05: significant, N: total number of patients, n: number of patients in the category. Data is presented in form of frequency (n) and percentage (%).

Parameters	Categories	Total number of patients N = 6732 (100%)		Toothache absent N = 2849 (42%)		Toothache present N = 3883 (58%)		p- value	Chi stat
		n	%	n	%	n	%		
Age (years)	Less than 17	2140	32%	919	43%	0.0001*	57%	0.0001*	44.50
	17 to 60	3180	47%	1234	39%	1946	61%		
	More than 60	1412	21%	696	49%	716	51%		
Sex	Male	2873	43%	1301	45%	1567	55%	0.0001*	18.94
	Female	3859	57%	1548	40%	2316	60%		
Diet	Veg	2987	44%	1251	42%	1736	58%	0.515	0.42
	Nonveg	3745	56%	1598	43%	2147	57%		
Smoking	Yes	1814	27%	670	37%	1144	63%	0.0001*	29.50
	No	4918	73%	2179	44%	2739	56%		
Tobacco	Yes	2312	34%	773	33%	1539	67%	0.000*	113.90
	No	4420	66%	2076	47%	2344	53%		
Alcohol	Yes	1418	21%	382	27%	1036	73%	0.000*	174.09
	No	5314	79%	2467	46%	2847	54%		
Brushing	Once	4829	72%	1801	37%	3028	63%	0.000*	176.60
	Twice	1903	28%	1048	55%	855	45%		
Dental Floss	Yes	630	9%	243	39%	387	61%	0.045*	4.00
	No	6102	91%	2606	43%	3496	57%		
Reported by	Self reported	1812	27%	561	31%	1251	69%	0.000*	1012.58
	Reported by spouse	2080	31%	459	22%	1621	78%		
	Reported by relatives	2840	42%	1829	64%	1011	36%		
Income per year	Below 2 lakhs	2035	30%	582	29%	1453	71%	0.000*	528.18
	2-4 lakhs	1528	23%	507	33%	1021	67%		
	4-6 lakhs	1699	25%	810	48%	889	52%		
	Above 6 lakhs	1470	22%	950	65%	520	35%		
History of medication	Self-medication	1922	29%	0	0%	1922	100%	0.000*	3317.28
	Medication by doctor	1077	16%	112	10%	965	90%		
	None	3733	55%	2735	73%	998	27%		
Residence	Rural	3990	59%	1429	36%	2561	64%	0.000*	169.85
	Urban	2742	41%	1420	52%	1322	48%		
Education	Illiterate	2156	32%	831	39%	1325	61%	0.0001*	72.06
	Less than primary	1613	24%	783	49%	830	51%		
	Up-to higher secondary	1748	26%	805	46%	943	54%		
	Graduate and above	1215	18%	430	35%	785	65%		
Reason for toothache	Caries	2222	33%	1212	43%	1010	26%	0.0001*	697.72
	Tooth fracture	1200	18%	670	24%	530	14%		
	Trauma	48	1%	0	0%	48	1%		
	Pericoronitis (Impacted tooth)	831	12%	148	5%	683	18%		
	Apical periodontitis	709	11%	167	6%	542	14%		
	Secondary caries	552	8%	312	11%	240	6%		
	Others	1170	17%	340	12%	830	21%		

The study evaluated factors associated with toothache among 3,883 patients, and significant associations were identified (p < 0.05). Age showed a significant link (p < 0.001), with the 17-60 years age group being the most affected, followed by those under 17 years and over 60 years, and female patients were more prevalent in the younger group. Diet was significantly associated (p = 0.027) with non-vegetarians (55%) reporting more toothaches, especially among female patients. Behavioral factors, such as smoking (p < 0.001), tobacco use (p < 0.001), and alcohol consumption (p < 0.001), were strongly linked, predominantly among male patients. Brushing (p = 0.009) and dental floss use (p < 0.001) were associated with a lower toothache prevalence, which was more common in females. Self-reported toothaches (p < 0.001) were higher among male patients, while spouses or relative reports were more frequent in female patients. Income showed a significant association (p < 0.001) with lower-income groups (<2 lakhs) reporting more toothaches, particularly female patients. The education level was significant (p < 0.001), with illiterate patients and those with higher education showing distinct patterns. The causes of toothache, including caries, pericoronitis, and apical periodontitis, were significantly associated (p < 0.001), with female patients being more affected by trauma and apical periodontitis. No significant association was found with residence (p = 0.511) (Table 2).

**Table 2:** Association of factors affecting toothaches between both sexes using the chi-square test of association. *p-value < 0.05: significant, N: total number of patients, n: number of patients in the category. Data are presented as frequency (n) and percentage (%).

Parameters	Categories	Total number of patients with toothache N= 3883 (100%)		Male N = 1567 (40.36%)		Female N = 2316 (59.64%)		p-value	Chi stats
		n	%	n	%	n	%		
Age (years)	Less than 17	1221	32%	412	34%	809	66%	0.000*	35.02
	17 to 60	1946	50%	826	42%	1120	58%		
	More than 60	716	18%	329	46%	387	54%		
Diet	Veg	1736	45%	734	42%	1002	58%	0.027*	4.83
	Non-veg	2147	55%	833	39%	1314	61%		
Smoking	Yes	1144	29%	827	72%	317	28%	0.000*	687.15
	No	2739	71%	740	27%	1999	73%		
Tobacco	Yes	1539	40%	927	60%	612	40%	0.000*	418.54
	No	2344	60%	640	27%	1704	73%		
Alcohol	Yes	1036	27%	824	80%	212	20%	0.000*	901.20
	No	2847	73%	743	26%	2104	74%		
Brushing	Yes	3028	78%	1255	41%	1773	59%	0.009*	6.80
	No	855	22%	312	36%	543	64%		
Dental Floss	Yes	387	10%	117	30%	270	70%	0.0001*	18.29
	No	3496	90%	1450	41%	2046	59%		
Reported by	Self-reported	1251	32%	812	65%	439	35%	0.000*	464.90
	Reported by spouse	1621	42%	445	27%	1176	73%		
	Reported by relatives	1011	26%	310	31%	701	69%		
Income per year	Below 2 lakhs	1453	37%	185	13%	1268	87%	0.000*	808.25
	2-4 lakhs	1021	26%	612	60%	409	40%		
	4-6 lakhs	889	23%	414	47%	475	53%		
	Above 6 lakhs	520	13%	356	68%	164	32%		
Medications	Self-medication	1920	49%	679	35%	1241	65%	0.000*	400.27
	Medication by doctor	965	25%	643	67%	322	33%		
	None	998	26%	245	25%	753	75%		
Residence	Rural	2561	66%	1024	40%	1537	60%	0.511	0.43
	Urban	1322	34%	543	41%	779	59%		
Education	Illiterate	1325	35%	196	15%	1129	85%	0.000*	7771.07
	Less than primary	830	21%	397	48%	433	52%		
	Up-to higher secondary	943	24%	678	72%	265	28%		
	Graduate and above	785	20%	296	38%	489	62%		
Reason for toothache	Caries	1010	26%	463	46%	547	54%	0.0001*	104.37
	Tooth fracture	530	14%	261	49%	269	51%		
	Trauma	48	1%	11	23%	37	77%		
	Pericoronitis (Impacted tooth)	683	18%	284	42%	399	58%		
	Apical periodontitis	542	14%	193	36%	349	64%		
	Secondary caries	240	6%	167	70%	73	30%		
	Others	830	21%	326	39%	504	61%		

Multivariate analysis of 3,883 patients investigated factors associated with toothache, revealing several significant associations. Age emerged as a key factor, with individuals aged 17 to 60 years showing 1.52 times higher odds of experiencing toothache compared to those under 17 years and those over 60 years showing 1.81 times higher odds, both statistically significant (p = 0.023 and p = 0.016, respectively). Lifestyle behaviors played a considerable role, where non-smokers had significantly higher odds of toothache than smokers (OR = 2.36, p = 0.021), and similar trends were noted among non-users of tobacco (OR = 1.98, p = 0.012) and non-alcohol users (OR = 2.12, p = 0.013), suggesting a possible reverse causality or reporting bias. Brushing and flossing practices were also significant; individuals who did not brush or floss had higher odds of toothache (OR = 1.05 and 1.96, respectively; p = 0.001 for both). Socioeconomic and demographic factors further influenced the outcomes. Urban residents were significantly more likely to report toothaches than rural residents were (OR = 1.5, p = 0.001). Education level showed a mixed trend; those with higher secondary education had significantly increased odds (OR = 0.72, p = 0.045), and graduates and above showed even higher odds (OR = 1.67, p = 0.001) than illiterate individuals. Income levels were also influential; individuals earning between 2 and 4 lakhs and above 6 lakhs had significantly different odds compared to those earning less than 2 lakhs (p = 0.045 for both), although ORs showed some inconsistencies with confidence intervals. The reporting source (self vs others) and medication history did not show statistically significant associations. Overall, the analysis highlighted the complex interplay of age, habits, education, income, and residence in determining the likelihood of toothache (Table 3).

**Table 3:** Multivariate analysis for factors associated with toothaches. *p-value < 0.05: significant. Ref: Reference category.

Parameters	Adjusted odds ratio among 3883 patients with toothaches				
	Categories	Odds ratio (OR)	Confidence interval at 95%		p-value
			Lower limit	Upper limit	
Age (years)	Less than 17	Ref			
	17 to 60	1.52	0.43	1.34	0.023*
	More than 60	1.81	0.76	2.45	0.016*
Diet	Vegetarian	Ref			
	Non-vegetarian	0.26	-0.81	1.34	0.068
Smoking	Yes	Ref			
	No	2.36	0.98	2.31	0.021*
Tobacco	Yes	Ref			
	No	1.98	0.86	3.67	0.012*
Alcohol	Yes	Ref			
	No	2.12	0.78	2.34	0.013*
Brushing	Yes	Ref			
	No	1.05	0.67	3.14	0.001*
Dental Floss	Yes	Ref			
	No	1.96	0.56	2.68	0.001*
Reported by	Self-reported	Ref			
	Reported by spouse	0.45	-1.26	3.45	0.129
	Reported by relatives	0.78	-1.87	3.21	0.324
Income per year	Below 2 lakhs	Ref			
	2-4 lakhs	0.04	-1.67	-1.32	0.045*
	4-6 lakhs	0.67	-1.34	-1.16	0.132
	Above 6 lakhs	1.04	0.76	2.31	0.045*
History of medication	Self-medication	Ref			
	Medication by doctor	0.78	-2.14	1.26	0.236
	None	1.03	-0.34	1.67	0.561
Residence	Rural	Ref			
	Urban	1.5	0.92	3.15	0.001*
Education	Illiterate	Ref			
	Less than primary	0.52	-1.31	2.56	0.081
	Up-to higher secondary	0.72	0.81	2.76	0.045*

Point-biserial correlation analysis for the etiological factors of toothaches revealed several significant associations. Caries showed a positive correlation with gender (0.45), brushing frequency (0.19), and self-medication (0.61), and a strong negative correlation with age (-0.34), education (-0.56), and income (-0.48). Tooth fracture was correlated with self-medication (0.56) and sex (0.08), while trauma was positively associated with self-medication (0.43) and self-reporting (0.21). Pericoronitis showed moderate correlation with sex (0.34), brushing (0.21), and self-medication (0.32). Periodontitis was strongly associated with age (0.56), sex (0.45), and self-report (0.37). Secondary caries had positive but weak correlations with smoking (0.03), brushing (0.16), and self-medication (0.23). Overall, self-medication had the highest correlation with toothache etiology among the various factors (Table 4).

**Table 4:** Point-biserial correlation (r) analysis for etiological factors for toothaches. r=-1: strong negative correlation, r= 0: no correlation, r=+1: strong positive correlation.

Factors	Caries	Fracture	Trauma	Impaction	Periodontitis	Secondary caries
Age	-0.34	-0.45	0.21	-0.34	0.56	0.13
Sex	0.45	0.08	0.05	0.34	0.45	0.23
Smoking	-0.21	0.02	0.05	0.12	0.23	0.03
Tobacco	-0.34	0.03	0.08	0.18	0.21	0.05
Alcohol	-0.12	0.02	0.08	0.13	0.18	0.09
Brushing	-0.19	0.13	0.18	-0.21	-0.16	-0.03
Dental Floss	-0.01	-0.01	-0.02	-0.02	-0.01	-0.01
Self-reporting	-0.36	0.18	0.21	0.21	0.37	0.12
Self-medication	0.61	0.56	0.43	0.32	0.32	0.23
Education	-0.56	0.16	0.02	0.01	0.18	-0.07
Income	-0.48	-0.01	-0.01	-0.03	-0.12	-0.08

## Discussion

This study was conducted on 6732 patients, of whom 58% reported experiencing a toothache, which is consistent with the high prevalence rates reported in previous studies [3,10]. The current study identified age, sex, lifestyle habits, and socioeconomic factors as significant predictors of toothaches. Age was found to be a significant factor, with younger patients aged < 17 years and those aged 17-60 years showing the highest prevalence of toothache. These findings align with earlier studies indicating that younger populations are more prone to dental caries and pulpitis owing to dietary habits and insufficient oral hygiene practices [3,11]. The observed association between younger individuals and increased toothache prevalence highlights the need for preventive strategies that focus on school-aged children and young adults.

The negative correlation between age and caries suggests that younger individuals are more susceptible to dental caries, likely due to the poorer oral hygiene practices prevalent in this age group [3,11]. In contrast, older individuals are less likely to report tooth fractures, which may be attributed to reduced physical activity and, thus, a lower risk of trauma-related injuries. Younger individuals are also more prone to pericoronitis, which is typically associated with the eruption of wisdom teeth. Conversely, the strong positive correlation between age and apical periodontitis indicates that older individuals are more susceptible to this condition, possibly due to accumulated dental issues and untreated infections progressing to periapical abscesses [12].

Sex disparities were also evident, with female patients reporting toothache more frequently than male patients across all age groups [13]. Female patients may be more likely to seek dental care and report symptoms earlier than their male counterparts. Additionally, hormonal differences and the impact of pregnancy on oral health could also contribute to the higher prevalence of toothaches in females [13]. A stronger positive correlation was observed between female sex and caries, potentially attributable to hormonal fluctuations during pregnancy, menstruation, or other physiological changes that increase susceptibility to oral diseases, including dental caries [14]. A moderate positive correlation with sex suggests that pericoronitis may be more prevalent in women, potentially due to hormonal influences that affect tissue responses around erupting teeth [14]. 

Lifestyle factors, including tobacco use, smoking, and alcohol consumption, were strongly associated with toothaches in the present study. This finding corroborates previous research linking poor lifestyle choices with higher rates of dental diseases such as caries and periodontal disease, which are primary contributors to toothache [11,12]. Smoking and tobacco use, in particular, have consistently been associated with gum disease and tooth loss, further aggravating dental pain [8]. The significant association between alcohol consumption and toothaches observed in this study may be due to its deleterious effects on oral tissues as well as lifestyle factors related to neglect of oral hygiene [15]. Smokers and tobacco users may be less inclined to seek dental care for minor issues, such as caries, leading to underreporting. Smoking and tobacco use had a moderate positive correlation with apical periodontitis. Smoking is a known risk factor for periodontal disease as it weakens the immune response and accelerates gum disease, which can lead to periapical infections [16].

Oral hygiene practices such as brushing frequency and flossing were also significantly associated with the prevalence of toothache. Patients who brushed less frequently and did not use dental floss were more likely to experience toothache [3,17]. These findings emphasize the critical role of maintaining good oral hygiene in preventing dental diseases. Similar findings have been reported in studies where insufficient brushing and flossing neglect were associated with a higher incidence of dental caries and periodontal issues [3,11]. A negative correlation with brushing frequency indicated that individuals who do not brush frequently report caries and periapical diseases. This suggests that brushing alone is insufficient, particularly if dietary habits or brushing techniques are inadequate.

Socioeconomic factors, including lower income, illiteracy, and rural residency, were significantly associated with toothache [3,9,11]. This reinforces the well-established relationship between socioeconomic status and oral health outcomes. Patients with lower income and education levels may have limited access to dental care services, further exacerbating the prevalence and severity of toothaches. These negative correlations suggest that individuals with higher education and income levels are less likely to suffer from caries. This is expected, as higher socioeconomic status is often associated with better health literacy, access to healthcare, and improved oral hygiene practices [9].

Furthermore, rural residents face barriers, such as inadequate healthcare infrastructure and a lack of awareness regarding oral hygiene, which contributes to the persistence of dental pain [9]. A strong positive correlation suggests that many individuals with caries attempt self-medication before seeking professional care. This might indicate a lack of access to dental services or a tendency to underestimate caries severity. Therefore, community-based programs should be implemented to increase awareness [18]. A positive correlation suggests that individuals with apical periodontitis are likely to report their condition and attempt to manage their symptoms using self-medication.

The findings of this study strongly support the existing literature that identifies dental caries and periapical infections as the most common causes of toothaches [3,19]. In our analysis, caries accounted for 43% of toothache cases, while apical periodontitis accounted for 14%, highlighting their significant role in dental pain. These conditions are the leading causes of toothache owing to their progressive nature, where untreated caries can lead to pulp inflammation and subsequent periapical infection [19]. Similar studies have consistently demonstrated that caries and periapical infections remain the primary etiological factors for dental pain, particularly in populations with poor oral hygiene practices [3].

Multivariate analysis in this study revealed that non-smokers, non-tobacco users, and non-alcohol consumers had higher odds of reporting toothache, which could be due to better self-reporting behaviors [20]. Moreover, the results showed that individuals with higher educational attainment and income had lower odds of toothache, which reflects the protective effects of socioeconomic advantages on oral health [9].

Clinical implications

The study underscores the need for targeted clinical interventions to address toothaches, particularly among younger patients and female patients, who reported higher prevalence across all age groups. Clinicians should prioritize early screening for dental caries and apical periodontitis, especially in females and lower-income individuals, where higher rates are linked to hormonal influences and limited healthcare access. The strong association of lifestyle factors, including smoking, tobacco use, and alcohol consumption, with toothache highlights the importance of oral health education to encourage the cessation of these habits. Promoting regular brushing and flossing is essential to reduce the incidence of toothache, particularly in populations with inadequate oral hygiene.

Limitations

Despite the valuable insights provided by this study, several limitations of this study should be acknowledged. First, the cross-sectional design limited the ability to establish causal relationships between the identified risk factors and toothaches. Second, self-reported data on toothaches and lifestyle factors such as alcohol and tobacco use might be subject to recall and social desirability biases, potentially influencing the accuracy of the findings. Finally, the study was conducted at a single center, which might not be representative of the general population, particularly in terms of access to care.

Future recommendations

Future research should focus on longitudinal studies to establish causal relationships between toothache and risk factors, such as age, sex, lifestyle, and socioeconomic status, addressing the limitations of the current cross-sectional design. Multicenter studies across diverse regions of India are recommended to enhance generalizability beyond single-center samples. Incorporating objective diagnostic tools, such as dental imaging, can reduce reliance on self-reported data and mitigate recall bias. Community-based oral health programs targeting rural residents and lower-income groups should be developed to improve access to dental care and raise awareness of effective oral hygiene practices. Additionally, exploring the role of hormonal influences on toothaches in women through targeted studies could inform sex-specific interventions.

## Conclusions

This study revealed a high prevalence of toothaches among patients, underscoring its critical public health significance in India. Toothaches are closely linked to demographic factors, including younger age, female sex, lower income, and limited education, as well as lifestyle factors such as smoking, tobacco use, and inadequate oral hygiene practices. Vulnerable groups, particularly women, rural residents, and those with socioeconomic disadvantages, face heightened risks. These findings emphasize the urgent need for targeted preventive strategies, including community-based educational programs, to promote effective oral hygiene and discourage harmful habits such as smoking and tobacco use. Addressing these diverse risk factors demands coordinated public health initiatives to enhance access to dental care, raise oral health awareness, and reduce the burden of toothaches, especially in underserved populations.

## Appendices

Questionnaire

Patient Identification

1. Name (Optional): ___________________________ 

2. Age: _______ years 

   - ☐ <17 

   - ☐ 17-60 

   - ☐ >60 

3. Gender: 

   - ☐ Male 

   - ☐ Female 

   - ☐ Other 

   - ☐ Prefer not to say 

Part 1: Demographic & Socioeconomic Details

4. Residence: 

   - ☐ Urban 

   - ☐ Rural 

5. Education Level: 

   - ☐ Illiterate 

   - ☐ Primary School 

   - ☐ Higher Secondary 

   - ☐ Graduate/Postgraduate 

6. Annual Income (INR/year): 

   - ☐ <2 lakh 

   - ☐ 2-4 lakh 

   - ☐ 4-6 lakh 

   - ☐ >6 lakh 

7. **Dietary Habits: 

   - ☐ Vegetarian 

   - ☐ Non-vegetarian 

Part 2: Toothache & Contributing Factors

8. Oral Hygiene Practices: 

   - Brushing frequency: ☐ Once daily ☐ Twice daily ☐ Rarely/Never 

   - Flossing: ☐ Yes ☐ No 

9. Habits: 

   - Smoking: ☐ Yes ☐ No 

   - Tobacco use (chewing/snuff): ☐ Yes ☐ No 

   - Alcohol consumption: ☐ Yes ☐ No 

10. Pain Details: 

    - Duration of current toothache: _______ days/weeks 

    - Pain intensity (VAS 0-10): ______ (0 = no pain, 10 = worst pain) 

11. Visit Details: 

    - Accompanied by: ☐ Self ☐ Spouse ☐ Relative/Friend ☐ Other 

    - Medication taken before visit: 

      ☐ Self-prescribed (e.g., painkillers) 

      ☐ Prescribed by doctor 

      ☐ None 

12. Suspected Cause of Toothache (Multiple options allowed): 

    ☐ Dental caries 

    ☐ Tooth fracture/crack 

    ☐ Gum disease (e.g., pericoronitis) 

    ☐ Trauma/injury 

    ☐ Apical periodontitis 

    ☐ Sensitivity 

    ☐ Other: _______________                  

Signature-

Date-

## References

[1] Cohen LA, Bonito AJ, Akin DR, Manski RJ, Macek MD, Edwards RR, Cornelius LJ (2009). Toothache pain: behavioral impact and self-care strategies. Spec Care Dentist.

[2] De Laat A (2020). Differential diagnosis of toothache to prevent erroneous and unnecessary dental treatment. J Oral Rehabil.

[3] Kakoei S, Parirokh M, Nakhaee N, Jamshidshirazi F, Rad M, Kakooei S (2013). Prevalence of toothache and associated factors: a population-based study in Southeast Iran. Iran Endod J.

[4] Peres MA, Macpherson LMD, Weyant RJ (2019). Oral diseases: a global public health challenge. Lancet.

[5] Kumar YS, Acharya S, Pentapati KC (2014). Prevalence of dental pain and its relationship to caries experience in school children of Udupi district. Eur Arch Paediatr Dent.

[6] García-Cortés JO, Mariel-Cárdenas J, Martinez-Rider R (2020). Dental pain and associated factors in Mexican adolescents and young adults: a cross-sectional study. Int Dent J.

[7] Jiang X, Jiang X, Wang Y, Huang R (2019). Correlation between tobacco smoking and dental caries: a systematic review and meta-analysis. Tob Induc Dis.

[8] Baskaradoss JK, Geevarghese A, Al-Mthen A, Al-Ghamdi H, Al-Haudayris R, Al-Obaidy S, Al-Saadi W (2019). Influence of lifestyle on dental health behavior. J Lifestyle Med.

[9] Shah N, Sundaram KR (2004). Impact of socio-demographic variables, oral hygiene practices, oral habits and diet on dental caries experience of Indian elderly: a community-based study. Gerodontology.

[10] Slade GD (2001). Epidemiology of dental pain and dental caries among children and adolescents. Community Dent Health.

[11] Kuhnen M, Peres MA, Masiero AV, Peres KG (2009). Toothache and associated factors in Brazilian adults: a cross-sectional population-based study. BMC Oral Health.

[12] Kaercher D, Thelen P, Ruettermann M, Li L, Hamprecht A (2024). Outcome predictors of odontogenic abscesses in the elderly. Front Oral Health.

[13] Lukacs JR, Largaespada LL (2006). Explaining sex differences in dental caries prevalence: saliva, hormones, and "life-history" etiologies. Am J Hum Biol.

[14] Sachelarie L, Iman AE, Romina MV, Huniadi A, Hurjui LL (2024). Impact of hormones and lifestyle on oral health during pregnancy: a prospective observational regression-based study. Medicina (Kaunas).

[15] Jansson L (2008). Association between alcohol consumption and dental health. J Clin Periodontol.

[16] Borojevic T (2012). Smoking and periodontal disease. Mater Sociomed.

[17] Marchesan JT, Byrd KM, Moss K (2020). Flossing is associated with improved oral health in older adults. J Dent Res.

[18] Nghayo HA, Palanyandi CE, Ramphoma KJ, Maart R (2024). Oral health community engagement programs for rural communities: a scoping review. PLoS One.

[19] Renton T (2011). Dental (odontogenic) pain. Rev Pain.

[20] Karem Hassan B, Jabbar Ali B, Mahmood Alwan A, Badeia RA (2020). Self-reported oral health attitudes and behaviors, and gingival status of dental students. Clin Cosmet Investig Dent.

